# Variability in Primary Care Physician Attitudes Toward Medicaid Work Requirement Exemption Requests Made by Patients With Depression

**DOI:** 10.1001/jamahealthforum.2021.2932

**Published:** 2021-10-01

**Authors:** Harald Schmidt, Andrew J. Spieker, Tianying Luo, Julia E. Szymczak, David Grande

**Affiliations:** 1 Department of Medical Ethics and Health Policy, Center for Health Incentives and Behavioral Economics, Perelman School of Medicine, University of Pennsylvania, Philadelphia; 2 Department of Biostatistics, Vanderbilt University Medical Center, Nashville, Tennessee; 3 Department of Computer and Information Sciences, Fordham University, Bronx, New York; 4 Perelman School of Medicine, Department of Biostatistics, Epidemiology and Informatics, University of Pennsylvania, Philadelphia; 5 Perelman School of Medicine, Division of General Internal Medicine, University of Pennsylvania, Philadelphia; 6 Leonard Davis Institute of Health Economics, University of Pennsylvania, Philadelphia

## Abstract

**Importance:**

Medicaid work requirements seek to promote health and personal responsibility but can also jeopardize health care access. Physicians have a central function in assisting patients with exemption requests, but it is unclear how their role affects patient welfare, professionalism, and the ethical and legal justification of programs.

**Objective:**

To understand the degree of variability in physician response to assist patients with depression in receiving a Medicaid work requirement exemption.

**Design, Setting, and Participants:**

We conducted a mailed survey experiment among practicing primary care physicians in the first 4 approved states (Arkansas, Kentucky, Indiana, New Hampshire) in July and October of 2019. We report response, cooperation, refusal, and contact rates in line with American Association for Public Opinion Research (AAPOR) standards.

**Exposures:**

In each state, we used an experimental factorial design to randomize recipients to 1 of 4 patient clinical scenarios.

**Main Outcomes and Measures:**

The primary outcome was the indicator of willingness to assist a patient reporting depression with an exemption.

**Results:**

We received 715 responses (overall AAPOR response rate: 21%; cooperation rate: 84%; refusal rate: 4%; contact rate: 25%). Respondents’ mean (SD) age was 54 (12) years; mean (SD) time since graduation, 26 (12) years; 435 (61%) identified as male; 177 as Democrat (25%); 156 as Republican (22%); 197 as Independent/other (28%); and 185 as declined/unknown (26%); the mean (SD) share of Medicaid patients was 29% (21%). We found that 97 of 387 physicians (25%) would offer assistance even when state policy would not support an exemption, and 170 of 315 (54%) would not offer assistance when regulations would require this. Moreover, 49 of 245 respondents (20%) who deemed an exemption appropriate indicated that they would not assist. State, administrative effort, political affiliation, and perceived appropriateness were statistically associated with the odds of assisting with an exemption.

**Conclusions and Relevance:**

In this survey study of primary care physicians, we found substantial variation regarding willingness to assist patients qualifying for a work requirement exemption where none should exist. Insofar as work requirements are implemented again, it is critical to proactively identify measures to ensure that patients qualifying for exemptions are not put at risk due to either the burdensomeness of exemption procedures, or physicians’ political or personal views.

## Key Points

**Question:**

To what degree do physicians vary in their willingness to assist patients seeking exemptions from Medicaid work requirements?

**Findings:**

In this mailed survey experiment with 715 responses, we found that 25% of physicians would assist a patient not qualifying under the state’s criteria for exemptions from Medicaid work requirements, and 54% would not, even when a patient qualifies. We found state, administrative effort, political affiliation, and perceived appropriateness to be significantly associated with willingness to assist.

**Meaning:**

It is medically, ethically, and legally imperative that measures aimed at protecting vulnerable patients are not undermined by the burdensomeness of exemption procedures, or by physicians’ political or personal views.

## Teaser

This experimental survey study examines variation in physician willingness to assist patients seeking exemptions from Medicaid work requirements.

## Introduction

Medicaid work and community engagement requirements (henceforth “work requirements”) make health care access conditional on reporting between 80 to 100 hours of qualifying activities per month, such as skills training, job searching, or informal caregiving. In January 2018, the Centers for Medicare & Medicaid Services (CMS) authorized states to test work requirements to promote “better mental, physical, and emotional health” and “to help individuals and families rise out of poverty and attain independence.”^1^ Critics, however, view the policy as incompatible with Medicaid’s most fundamental objective: to furnish health care access.^2,3,4,5,6,7,8^ Most research has focused on the justification of policies and the ability of beneficiaries to comply with program requirements.^2,3,4,5,6,7,8,9,10,11,12^ The role of physicians’ behavior merits similar attention: policies in all states assign them an integral role in assisting patients with requests to be exempted, and in doing so, require them to exercise considerable judgment.

Work requirements have been challenged in the courts by Medicaid beneficiaries. After the US Court of Appeals for the District of Columbia Circuit affirmed rulings in favor of the plaintiffs, the Trump administration appealed the ruling. The US Supreme Court had initially agreed to hear the cases of Arkansas and New Hampshire in Spring 2020. However, at the request of the Biden administration, the Court canceled an already scheduled hearing.^13,14^ As of this writing, work requirements had been approved in 6 states, blocked by the courts in 4 and were halted in 2, whereas 7 applications remained submitted to CMS (eTable 1 in the Supplement). In April 2021, CMS notified Arizona, Arkansas, Indiana, Michigan, New Hampshire, and Wisconsin of its final decision to withdraw work requirement waiver authorities,^15^ though states may appeal this decision and it is possible that the Supreme Court will still hear the case in the next term.^16^ Although it is clear that the Biden administration is opposed to work requirements, future administrations could authorize the initiative again; a full assessment of the policy’s effect on different stakeholders hence remains critical and relevant.

All approved work requirement programs recognized that some life circumstances can render compliance unduly burdensome for some groups: full time students, pregnant women, or individuals with severe disabilities, for instance, are routinely exempt, subject to verification by health professionals or other relevant parties.^11,12^ Moreover, each state established additional medical frailty exemptions^12^ that may be initiated by patients but, at different points in the process, uniformly require a physician’s attestation. However, procedural arrangements differ across states. Variation exists, for example, in administrative burden and the extent to which a physician’s certification by itself is sufficient to receive an exemption. eTable 1 in the Supplement shows a schematic overview of the processes in the first 4 approved states.

Medical frailty exemptions matter because research has shown that most beneficiaries either already meet work requirements or would qualify for such exemptions.^2,9,11,12,17,18,19^ However, because exemptions require administrative effort, physicians might be reluctant to assist even when beneficiaries might be harmed as a consequence. Evaluations of prior related reforms suggested that reluctance to request exemptions led to a 10% overall increase in avoidable emergency department use.^20^

There is also evidence that political affiliation of physicians varies by geography and specialty,^21,22^ and that physician behavior is influenced by political views or personal attitudes.^23,24^ Physician decisions could increase risks of harm to beneficiaries, or, conversely, lead to beneficiaries being exempt even if, on regulatory intent, they should not be exempt.^25^ Acting in nonconcordant ways may create tensions for physicians as they navigate competing personal, professional, and organizational values.^26^ Further, nonconcordant behavior risks compromising program evaluations, as possible benefits—or, more problematically, harms—may be underestimated. Understanding possible variability therefore matters for patient welfare, physician professionalism, evidence about the effectiveness of programs, as well as ethically and legally.

## Methods

### Design

We conducted a survey experiment among practicing primary care physicians (PCPs) to understand (1) the degree of uniformity with which PCPs respond in implementing medical frailty exemptions, and (2) insofar as there might be variations, what can explain them. We mailed a 1-page cover letter, survey instrument (4 pages), and brief background information on each state’s work requirements provisions and exemption processes, using reproductions of the state’s information for the public and clinicians (ranging by state from 4-12 pages). Where email addresses were available, recipients also received an electronic invite to view the same information about the policy features online, and to complete the survey on a computer, tablet, or phone. The initial survey was sent July 15, 2019; after 3 reminders, all remaining PCPs with unconfirmed dispositions were contacted again October 4, 2019. We focused on depression as, compared to the general population, prevalence is markedly higher in low-income Americans, with around 30% having been diagnosed with it.^27^ Prior research also suggests that views on depression, although often associated with stigma, are not associated directly with political partisanship.^23^ This study was approved by the University of Pennsylvania institutional review board (protocol #832562). Survey participants provided implied consent by returning the survey by mail or completing it online (data use arrangements were detailed in all versions of the instrument).

### Sample

We obtained a sample of mail and email addresses of practicing PCPs accepting Medicaid patients in the first 4 approved states (Arkansas [AR], Kentucky [KY], Indiana [IN], New Hampshire [NH]) from SK&A/IQVIA, comprising 5561 individuals. In light of study resources and power calculations, we fielded to 100% of PCPs in AR and NH but selected a random subset of 80% of PCPs in the 2 larger states (IN, KY), yielding a total of 4160 PCPs (AR = 923, IN = 1332, KY = 1331, NH = 574).

### Instrument and Experimental Design

Using a 2×2 factorial experimental design, we randomized recipients in each state to respond to a patient clinical scenario. The clinical scenario varied in the disease severity (minor vs major depression) and duration of the physician-patient relationship (new patient vs having been seen for 2 years). On both *International Statistical Classification of Diseases and Related Health Problems, Tenth Revision (ICD-10)* and *Diagnostic and Statistical Manual of Mental Disorders* (Fifth Edition), diagnosing a major depressive disorder is appropriate if 5 or more of 9 overall symptoms are present during the same 2-week time period that represents changes in functioning, and if at least 1 symptom is either a depressed mood or loss of interest.^28^ The minor depression vignette included 3 of 9 symptoms, not triggering an exemption in any of the 4 states. The major depression vignette included 7 of 9 symptoms. In all 4 states, on regulatory intent, this condition would either be sufficient by itself to trigger an exemption review or play a major role. The vignette also stated that the beneficiary told the PCP that he had heard he could be exempted because of his depression, that a friend gave him the state’s information sheet (included in the survey letter) and that, should he receive no exemption, he would lose Medicaid coverage in about 3 weeks, due to not meeting work requirements (eMethods in the Supplement). Physician participants were asked whether they would assist the patient with an exemption. Respondents were also asked to rate the appropriateness of the exemption and the administrative effort required to provide it. Separately, we elicited baseline knowledge about the exemption process, PCP perception of the likelihood that they would encounter patients who lost coverage due to the policy, and overall approval of the policy. Two free-text fields enabled PCPs to share additional thoughts. We also captured 9 personal, professional, and practice characteristics (Table 1).

**Table 1.** Descriptive Statistics of Demographic Characteristics, Overall, and Stratified by State

Characteristic	Primary care physicians, No. (%)				
	All	Arkansas	Indiana	Kentucky	New Hampshire
No.	715	144	268	197	106
Age, y					
28-45	186 (26.0)	34 (23.6)	79 (29.5)	55 (27.9)	18 (17.0)
46-55	164 (22.9)	30 (20.8)	56 (20.9)	46 (23.4)	32 (30.2)
56-65	194 (27.1)	36 (25.0)	72 (26.9)	53 (26.9)	33 (32.1)
66-111	109 (15.2)	19 (20.1)	36 (13.4)	30 (15.2)	14 (13.2)
Unknown	62 (8.7)	15 (10.4)	25 (9.3)	13 (6.6)	9 (8.5)
Gender					
Female	225 (31.5)	33 (22.9)	94 (35.1)	61 (31.0)	37 (34.9)
Male	435 (60.8)	96 (66.7)	154 (57.1)	123 (62.4)	63 (59.4)
Other/declined^a^	10 (1.4)	1 (0.7)	5 (1.9)	3 (1.5)	1 (0.9)
Unknown	45 (6.3)	14 (9.7)	16 (6.0)	10 (5.1)	5 (4.7)
Race and ethnicity					
Asian	54 (7.6)	8 (5.6)	25 (9.3)	19 (9.6)	2 (1.9)
Hispanic	11 (1.5)	2 (1.4)	5 (1.9)	2 (1.0)	2 (1.9)
Non-Hispanic					
White	528 (74.0)	103 (71.5)	182 (67.9)	154 (78.2)	90 (84.9)
Black	23 (3.2)	9 (6.2)	11 (4.1)	2 (1.0)	1 (0.9)
Other/declined^b^	56 (7.8)	9 (6.2)	30 (11.2)	11 (5.6)	6 (5.7)
Unknown	42 (5.9)	13 (9.0)	15 (5.6)	9 (4.6)	5 (4.7)
Political affiliation					
Democrat	177 (24.8)	27 (18.8)	53 (19.8)	49 (24.9)	48 (45.3)
Republican	156 (21.8)	28 (19.4)	64 (23.9)	56 (28.4)	8 (7.6)
Independent/other	197 (27.6)	48 (33.3)	77 (28.7)	39 (19.8)	33 (31.1)
Declined	137 (19.2)	28 (19.4)	58 (21.6)	40 (20.4)	11 (10.4)
Unknown	48 (6.7)	13 (9.0)	16 (6.0)	13 (6.6)	6 (5.7)
Time since medical school graduation, y					
0-15	138 (19.3)	25 (17.4)	53 (19.8)	46 (23.4)	14 (13.2)
16-25	165 (23.1)	28 (19.4)	74 (27.6)	34 (17.3)	29 (27.4)
26-35	171 (23.9)	30 (20.8)	56 (20.9)	53 (26.9)	32 (30.2)
36-59	148 (20.7)	35 (24.3)	52 (19.4)	42 (21.3)	19 (17.9)
Unknown	93 (13.0)	26 (18.1)	33 (12.3)	22 (11.2)	12 (11.3)
Specialty					
Internal	161 (22.5)	19 (13.2)	47 (17.5)	65 (33.0)	30 (28.3)
General	466 (65.2)	102 (70.8)	189 (70.5)	109 (55.3)	66 (62.3)
Family	14 (2.0)	6 (4.2)	5 (1.9)	3 (1.5)	0 (0.0)
Other	28 (3.9)	4 (2.8)	11 (4.1)	10 (5.1)	3 (2.8)
Unknown	46 (6.4)	13 (9.0)	16 (6.0)	10 (5.1)	7 (6.6)
Medicaid patients, %					
0-15	210 (29.5)	35 (24.7)	104 (38.8)	39 (19.8)	32 (30.5)
16-25	122 (17.1)	29 (20.4)	36 (13.4)	32 (16.2)	25 (23.8)
26-35	145 (20.4)	30 (21.1)	46 (17.2)	44 (22.3)	25 (23.8)
36-96	176 (24.7)	33 (23.2)	59 (22.0)	69 (25.0)	15 (14.3)
Unknown	59 (8.3)	15 (10.6)	23 (8.6)	13 (6.6)	8 (7.6)
Physicians in practice					
1	133 (18.6)	43 (29.9)	39 (14.6)	41 (20.8)	10 (9.4)
2-10	368 (51.5)	62 (43.1)	143 (53.4)	114 (57.9)	49 (46.2)
11-50	122 (17.1)	21 (14.6)	44 (16.4)	24 (12.2)	33 (31.1)
≥51	47 (6.6)	4 (2.8)	26 (9.7)	8 (4.1)	9 (8.5)
Unknown	45 (6.3)	14 (9.7)	16 (6.0)	10 (5.1)	5 (4.7)

^a^ “Other” category includes “declined” and those who wrote in a gender other than male/female.

^b^ “Other” category includes “declined,” American Indian, Native Hawaiian/Pacific Islander, Other, and mixed.

### Outcomes

The primary outcome was the indicator of willingness to assist a patient seeking an exemption due to medical frailty. Secondary outcomes included an ordinal measure of attitude toward appropriateness of exemption.

### Statistical Analysis

All analyses were conducted in R statistical software (version 4.0.3, R Foundation). Descriptive statistics were determined both overall and stratified by state for age, gender, race and ethnicity, political affiliation, time since graduation, medical specialty, proportion of Medicaid patients, and number of physicians in the respondent's practice. Analytically, continuous variables were not categorized; however, to report statistics uniformly as absolute and relative frequencies, we treated continuous variables categorically. To address missing data in all regression-based analyses, we used multiple imputation and in each model, a corresponding robust variance estimator to accommodate possible model misspecification.^29^ Statistical significance was determined to be achieved at the nominal α = .05 level (2-sided).

To investigate factors predicting whether a PCP would seek to assist with an exemption, we fit a logistic regression model with binary vignette response as the outcome using the following predictors: age, gender, race/ethnicity, state, political affiliation, time since graduation, medical specialty, proportion of Medicaid patients, number of physicians in practice, duration scenario, and attitude regarding administrative burden. To determine predictors of perceived appropriateness of exemption, we used an analogous proportional odds model (ie, using the same predictors as stated) categorizing the outcome in ascending order of appropriateness. We further conducted complete-case analyses for regression models as a sensitivity analysis.

## Results

### Study Population

A total of 715 complete responses were received, yielding, by American Association for Public Opinion Research standards,^30^ an average overall response rate (RR) of 20.9% (RR1, 20.6%; RR2, 21.2%; RR3, 20.6%; RR4, 21.2%), average cooperation rates (CRs) of 83.8% (CR1, 82.6%; CR2, 84.9%; CR3, 82.6%; CR4, 84.9%) refusal rate of 3.8% (RefR, 1-3 all identical) and a contact rate of 24.9% (ContR, 1-3 all identical). Respondents’ mean (SD) age was 54 (12) years; mean (SD) time since graduations from medical school was 26 (12) years; 435 of 715 (61%) identified as male, 177 as Democrat (25%), 156 as Republican (22%), 197 as Independent or other (28%), and 185 respondents declined or were unknown (26%). The mean (SD) share of Medicaid patients was 29% (21%). Descriptive statistics on demographic factors are shown in Table 1. Nonresponse bias analyses were conducted on variables for which data on responders and the pool of contacted individuals were available, and demonstrated only modest differences: The overall standardized mean difference in age and time since graduation was less than 0.4 years; for categorical variables (gender, specialty, physicians in practice, Area Deprivation Index of practice location), the difference in proportions between responders and the different types of nonresponders never exceeded 4.4% (eResults and eTables 2-4 in the Supplement).

### Physician Willingness to Assist With a Medical Frailty Exemption Request

**Figure 1.**
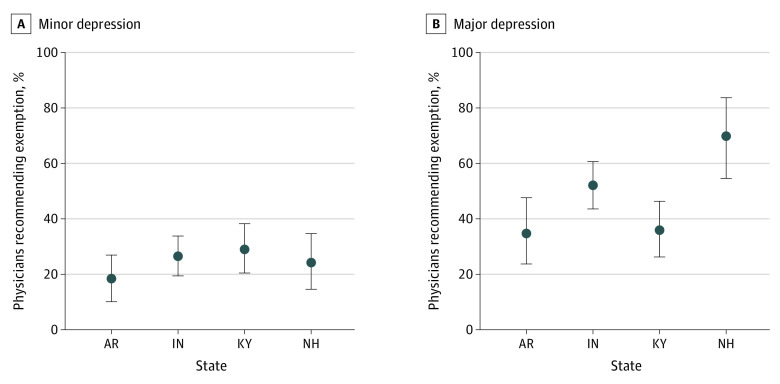
Point Estimates and 95% Confidence Intervals for Proportions Responding That They Would Seek an Exemption for the Patient, Stratified by Vignette Scenario (Minor or Major Depression), and Further by State Abbreviations: AR, Arkansas; IN, Indiana; KY, Kentucky; NH, New Hampshire.

Across the 4 states, counter to regulatory intent, 97 of 387 (25.1%) of PCPs randomized to the minor depression scenario would assist with a medical frailty exemption. Among PCPs randomized to the severe depression, 145 of 315 (46.0%) indicated that they would assist the patient seek an exemption, indicating that most are not complying with regulatory intent. We observed little variation across the 4 states in the minor depression scenario. However, there was substantial variation in the major depression scenario, ranging from 22 of 63 (34.9%) PCPs indicating that they would assist (AR) to 27 of 39 (69.2%; NH); point estimates and corresponding 95% confidence intervals are depicted in Figure 1 across states for each scenario. We did not find sufficient evidence of an association between PCP willingness to assist with an exemption and described length of the clinical relationship. Adjusted for state, those randomized to the longer duration scenario had an estimated 4.2% higher odds of indicating willingness to assist with an exemption as compared with those randomized to the shorter duration scenario (95% CI, −24.0% to 42.9%; *P* = .80).

### Factors Associated With PCP Willingness to Assist When Indicated

Regarding factors associated with assisting with an exemption, we focus on respondents randomized to the major depression scenario, due to the higher stakes for beneficiaries. We hypothesized that several factors may be associated with PCP’s decisions–respondent views related to administrative burden, practice characteristics such as the practice size (larger practices may have access to more administrative or social work support for administrative requests), Medicaid share of patients in the practice, state (given that states’ procedures vary), and political affiliation.

A multivariable model provided strong evidence of an association between state (omnibus *P* value: *P* = .01), political affiliation (omnibus *P* value: *P* = .002), and perceived administrative effort (omnibus *P* value: *P* < .001), and odds of assisting with an exemption. Specifically, NH respondents had the highest odds of indicating willingness to assist. Republicans had an estimated 74.5% lower odds of indicating willingness to assist, compared with Democrats (95% CI, 42.7%-88.7%; *P* = .001), and Independents/others had an estimated 61.2% lower odds (95% CI, 18.2%-81.6%; *P* = .01). Those indicating administrative effort as being more appropriate had 313% higher odds of indicating willingness to assist, compared with those viewing administrative effort as highly inappropriate (95% CI, 59.9%-514%; *P* < .001). Table 2 reports the estimated adjusted odds ratios and their respective confidence intervals and *P* values for all variables included in this model. Our complete-case sensitivity analysis involved 266 complete cases (out of 321 randomized to the major depression scenario); no conclusions from this sensitivity analysis were found to differ from the main results.

**Table 2.** Factors Associated With Physician Willingness to Assist Patients With Major Depression (1 = yes; 0 = no), Among Respondents Randomized to the Major Depression Scenario^a^

Variable	OR (95% CI)	*P* value
Age, y	1.03 (0.98-1.082)	.32
Gender		
Female	1 [Reference]	1 [Reference]
Male	0.81 (0.45-1.47)	.50
Other/declined	0.68 (0.09-5.13)	.71
Race and ethnicity		
Non-Hispanic		
White	1 [Reference]	1 [Reference]
Black	1.64 (0.31-8.54)	.56
Hispanic	1.25 (0.43-3.58)	.68
Asian	0.80 (0.15-4.34)	.79
Other/declined	0.59 (0.21-1.60)	.30
State		
Arkansas	1 [Reference]	1 [Reference]
Indiana	1.98 (0.97-4.07)	.06
Kentucky	1.05 (0.49-2.25)	.90
New Hampshire	3.86 (1.48-10.0)	.006
Political affiliation		
Democrat	1 [Reference]	1 [Reference]
Republican	0.26 (0.11-0.57)	.001
Independent/other	0.39 (0.18-0.83)	.01
Declined	0.92 (0.40-2.08)	.83
Graduated, y	0.99 (0.93-1.04)	.64
Specialty		
Internal	1 [Reference]	1 [Reference]
General	0.99 (0.51-1.92)	.97
Family	0.35 (0.04-2.88)	.33
Other	1.13 (0.30-4.24)	.86
Medicaid patients, %	0.99 (0.98-1.01)	.53
Physicians in practice		
1	1 [Reference]	1 [Reference]
2-10	1.06 (0.48-2.36)	.88
11-50	2.37 (0.90-6.28)	.08
≥51	0.72 (0.20-2.60)	.61
Duration		
Shorter	1 [Reference]	1 [Reference]
Longer	1.22 (0.71-2.09)	.48
Administrative effort		
Inappropriate	1 [Reference]	1 [Reference]
Neutral	3.13 (1.60-6.14)	<.001
Appropriate	4.42 (2.36-8.30)	<.001

Abbreviation: OR, odds ratio.

^a^ Sensitivity analysis included informedness as a covariate and did not find evidence of an association with odds of assisting.

**Figure 2.**
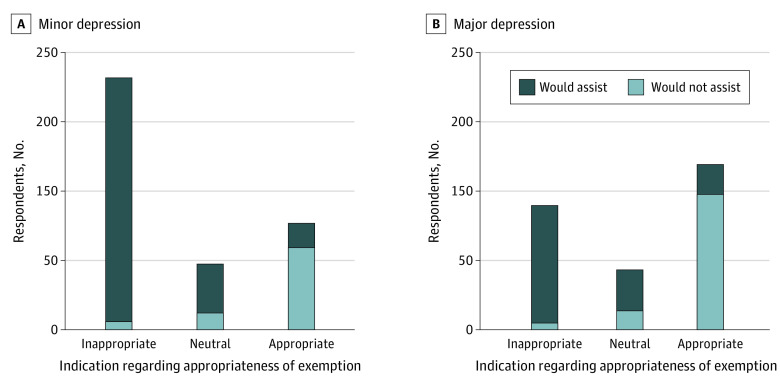
Concordance Between Attitude Regarding Appropriateness of Exemption and Indication of Willingness to Assist, Stratified by Severity Group (Minor vs Major Depression)

As a descriptive exploratory analysis, Figure 2 illustrates the degree to which responses regarding appropriateness of exemption are concordant with responses regarding willingness to assist. A total of 49 of 245 (20.0%) of respondents indicating that an exemption would be appropriate also indicated that they would not assist with an exemption. Of further note in this subset, 34 of 49 (69.4%) indicated that they believed the administrative effort to be either inappropriate or completely inappropriate, compared with 382 (54.3%) of the overall sample.

Overall PCP attitudes regarding appropriateness of an exemption in the major depression scenario varied by state (omnibus *P* value: *P* = .03). Respondents in KY were least likely to find the scenario appropriate for exemption, whereas those in NH were most likely. Republicans had a 79.7% lower odds of reporting a higher degree of exemption appropriateness compared with Democrats (95% CI, 60.8%-89.5%; *P* < .001); Independents were estimated to have a 65.2% lower adjusted odds compared with Democrats (95% CI, 30.5%-82.6%; *P* = .003). Further, those finding the administrative effort to be appropriate were estimated to have a 115% higher adjusted odds of reporting a higher degree of exemption appropriateness compared with those finding the administrative effort to be inappropriate (95% CI, 23.6%-273%; *P* = .007). Further results from this model are shown in eTable 5 in the Supplement.

### Physicians’ Perceptions of Work Requirement Policies

Respondents were asked to indicate how informed they were about the policy, whether the administrative effort was appropriate, and whether they approved of the policy. The results are presented in Table 3. With little variation across states, 383 of 708 respondents reported feeling uninformed about their role in exemption processes (54.1%), compared with 139 who reported feeling informed (19.6%). Similarly, 382 of 704 (54.2%) felt that the administrative effort was inappropriate, again, with little variation across states. However, 419 of 715 respondents approved of the work requirement policy (58.6%), with particularly high approval in IN (172/260 [64.2%]), and particularly low approval in NH (41/105 [38.7%]).

**Table 3.** Descriptive Statistics of Demographic Characteristics, Overall, and Stratified by State

Characteristic	Primary care physicians, No. (%)				
	All	Arkansas	Indiana	Kentucky	New Hampshire
No.	715	144	268	197	106
Informedness regarding role					
Fully	31 (4.3)	6 (4.2)	13 (4.9)	11 (5.6)	1 (0.9)
Sufficiently	108 (15.1)	25 (17.4)	42 (15.7)	27 (13.7)	14 (13.2)
Somewhat	186 (26.0)	37 (25.7)	70 (26.1)	51 (25.9)	28 (26.4)
Insufficiently	257 (35.9)	51 (35.4)	96 (35.8)	65 (33.0)	45 (42.5)
Fully uninformed	126 (17.6)	24 (16.7)	45 (16.8)	40 (20.3)	17 (16.0)
Unknown	7 (1.0)	1 (0.7)	2 (0.8)	3 (1.5)	1 (0.9)
Administrative effort					
Appropriate					
Completely	57 (8.0)	15 (10.4)	19 (7.1)	14 (7.1)	9 (8.5)
Somewhat	124 (17.3)	17 (11.8)	56 (20.9)	31 (15.7)	20 (18.9)
Neutral	141 (19.7)	34 (23.6)	60 (22.4)	32 (16.2)	15 (14.2)
Inappropriate					
Somewhat	189 (26.4)	35 (24.3)	72 (26.9)	56 (28.4)	26 (24.5)
Completely	193 (27.0)	41 (28.5)	54 (20.2)	63 (32.0)	35 (33.0)
Unknown	11 (1.5)	2 (1.4)	7 (2.6)	1 (0.5)	1 (0.9)
Overall approval of work requirement					
Approve					
Strongly	225 (31.5)	40 (27.8)	101 (37.7)	68 (34.5)	16 (15.1)
Somewhat	194 (27.1)	50 (34.7)	71 (26.5)	48 (24.4)	25 (23.6)
Neutral	96 (13.4)	14 (9.7)	46 (17.2)	18 (9.1)	18 (17.0)
Disapprove					
Somewhat	80 (11.2)	17 (11.8)	23 (8.6)	24 (12.2)	16 (15.1)
Strongly	107 (15.0)	23 (16.0)	19 (7.1)	35 (17.8)	30 (28.3)
Unknown	13 (1.8)	0	8 (3.0)	4 (2.0)	1 (0.9)

## Discussion

In this survey study of PCPs, we obtained responses from more than 700 PCPs practicing in the first 4 states approved to test Medicaid work requirements. We found that 25% would offer assistance even when the state’s medically frail criteria would not support an exemption, and 54% would not offer assistance when a patient does quality for assistance. We found that 20% of respondents who deem an exemption appropriate indicated that they would not assist a patient with requesting one. We further noted substantial variation by state, and that political affiliation, administrative effort, and perceived appropriateness were statistically associated with the odds of assisting with an exemption and can explain heterogeneity in physician decisions where none should exist.

In line with earlier research, we find that physicians are unwilling to simply implement in mechanistic ways rules that conflict with their personal and professional preferences, and that partisan bias can lead to unwarranted variation in patient care.^23,31^ From a patient welfare perspective, our findings have both upsides and downsides.

On the one hand, a quarter of PCPs signal that they would assist with an exemption even if regulations would not permit this. This aligns with position statements of major medical associations that expressed major concern about the likelihood that the work requirements jeopardize health care access^32^ and prior attitudinal and empirical research suggesting that between 10% to 60% of physicians will find workarounds to ensure that patients receive needed care, even when they risk disciplinary sanctions (for example, by exaggerating the severity of conditions; changing billing diagnoses; and/or reporting signs or symptoms patients did not have, absorbing cost of treatment, actively referring patients to no-fee safety-net physicians, or violating duty hour restrictions).^33,34,35,36,37,38,39,40,41,42^

On the other hand—and far more concerning—half of PCPs indicate that they would not assist a patient even when regulations suggest that they should do so, to mitigate the risk of harming patients by revoking health care access.

In terms of implications for ongoing legal challenges that center on whether work requirements are incompatible with Medicaid’s objective of furnishing health care access, our study highlights that there is a need to address the fact that even when work requirement’s procedures include rules seeking to protect beneficiaries, variability in PCP’s behavior can directly harm beneficiaries. Our findings suggest that the administrative burden of exemption procedures is regarded as inappropriately high by many PCPs, and that it may cause a PCP to not seek an exemption even if it is warranted. Insofar as policies such as work requirements should be deemed acceptable by the Supreme Court or are otherwise implemented again, it is critical that CMS proactively identify measures to ensure that patients qualifying for exemptions are not put at risk due to either the burdensomeness of exemption procedures, or physicians’ political or other views.

### Strengths and Limitations

In absolute terms, our response rate was low. However, we have sufficient statistical power to support our central conclusions and the experimental design provides strong internal validity in a balanced sample, including political affiliation. We reported stated, not revealed preferences, and social desirability bias can convey a rosier picture, moreover, nonresponse bias analyses revealed only modest differences. Although generally a limitation, in our case this only strengthens concerns about the actual effect of nonconcordant behavior because respondents could easily have made statements in line with regulatory intent. The condition used in the vignette was depression: results may differ for other conditions. Yet, given the noted high prevalence among the Medicaid population,^36^ the focus is certainly meaningful. Frailty determinations are only 1 way in which PCPs are involved in the process of deciding who is expected to meet work requirements (others are disability status determinations, and related issues arise routinely in sick-leave notes and other decisions). Medical frailty determinations therefore are not singularly unique, but still matter centrally, given the large number of eligible beneficiaries.^12^

## Conclusions

Work requirements established new obligations for both beneficiaries and physicians. Although concerns about the demandingness of exemption procedures are entirely understandable, the willingness to assist beneficiaries qualifying for medical frailty exemptions is also determined by political views, and the perceived appropriateness of exemptions. Work requirements are not the only case where personal preferences can make a difference between vulnerable populations retaining health care access or not, but the effect of these preferences needs to be considered more fully in program design and evaluations, as well as in the ongoing review of the legal justification for the policy.

## Notes

Supplement. eTable 1. Work requirements in the first 4 approved states:* options for exemptions from compliance due to medical frailty for Medicaid beneficiaries and primary care physicians eMethods. Sample survey instrument and description eResults. Non-response bias analysis eTable 2. Comparison of responders to all remaining eligible recipients (overall, and by state) eTable 3. Comparison of responders to decliners (overall, and by state) eTable 4. Comparison of responders to unconfirmed dispositions (overall, and by state) eTable 5. Proportional odds model regarding appropriateness of exemption
