# Supplementary material for: Variability in Primary Care Physician Attitudes Toward Medicaid Work Requirement Exemption Requests Made by Patients With Depression
Source: JAMA Health Forum. 2021 Oct 1;2(10):e212932. doi: 10.1001/jamahealthforum.2021.2932 (PMC8727036; doi:10.1001/jamahealthforum.2021.2932)
Supplement: Supplement. — eTable 1. Work requirements in the first 4 approved states:* options for exemptions from compliance due to medical frailty for Medicaid beneficiaries and primary care physicians eMethods. Sample survey instrument and description eResults. Non-response bias analysis eTable 2. Comparison of responders to all remaining eligible recipients (overall, and by state) eTable 3. Comparison of responders to decliners (overall, and by state) eTable 4. Comparison of responders to unconfirmed dispositions (overall, and by state) eTable 5. Proportional odds model regarding appropriateness of exemption [file jamahealthforum-e212932-s001.pdf]

Supplemental Online Content

Schmidt H, Spieker AJ, Luo T, Szymczak JE, Grande D. Variability in primary care physician attitudes toward Medicaid work requirement exemption requests made by patients with depression. *JAMA Health Forum*. 2021;2(10):e212932. doi:10.1001/jamahealthforum.2021.2932

eTable 1. Work requirements in the first 4 approved states:* options for exemptions from compliance due to medical frailty for Medicaid beneficiaries and primary care physicians
eMethods. Sample survey instrument and description
eResults. Non-response bias analysis
eTable 2. Comparison of responders to all remaining eligible recipients (overall, and by state)
eTable 3. Comparison of responders to decliners (overall, and by state)
eTable 4. Comparison of responders to unconfirmed dispositions (overall, and by state)
eTable 5. Proportional odds model regarding appropriateness of exemption

This supplemental material has been provided by the authors to give readers additional information about their work.

eTable 1: Work requirements in the first 4 approved states:[*] options for exemptions from compliance due to medical frailty for Medicaid beneficiaries and primary care physicians

Procedural arrangements differ across states. While patients can initiate the exemption process in all four states, physicians' attestations can strengthen an application, are sometimes required, and would typically be ordered by the administrative unit evaluating the exemption request at a later point. In overall process-terms, variation exists, for example, in the entailed administrative burden and the extent to which a physician's certification by itself is likely to be sufficient for receiving an exemption: a schematic overview of the processes in the first four approved states is provided below, and further detail on Arkansas' arrangements are provided in the sample instrument on page 3ff (details on other states' processes were provided to survey respondents accordingly, and are available from the corresponding author).

	Kentucky[#]	Indiana	Arkansas	New Hampshire
**Can PCP grant exemption?**	No	No	No	(Yes)
**What can PCP do?[^]**	1. Complete medical frailty certification (7 pages, 22 questions, with condition codes to be added from separate 10 page document), or<br>2. Assist with self-attestation (informal letter).	1. Assist with self-attestation (informal letter).	1. Become a Registered Reporter (view 2 instructional videos, complete 1 page form), and<br>2. Complete medical frailty certification in online portal (not publicly available), or<br>3. Assist with self-attestation (informal letter).	1. Complete medical frailty certification (2 pages, 5 questions), or<br>2. Assist with self-attestation (informal letter).
**Ultimate determination**	Managed Care Organization	Managed Care Organization	Managed Care Organization	Health Department (though it is understood that the HD follows the PCP's recommendation without further scrutiny)

* As of July 20, 2021, the status of work requirements was as follows: 6 approved (Arizona, Georgia, Nebraska, Ohio, South Carolina, Wisconsin) , 4 blocked by the courts (Arkansas, Kentucky, Michigan, New Hampshire); 2 halted (Utah, Indiana); while 7 applications remained submitted to CMS (Alabama, Idaho, Mississippi, Montana, Oklahoma, South Dakota, Tennessee). In April 2021, CMS notified Arizona, Arkansas, Indiana, Michigan, New Hampshire, and Wisconsin of its final decision to withdraw work requirement waiver authorities, though states may appeal this decision. See: The Commonwealth Fund. Status of Medicaid Expansion and Work Requirement Waivers, July 20, 2021, available at: https://www.commonwealthfund.org/publications/maps-and-interactives/2021/jul/status-medicaid-expansion-and-work-requirement-waivers. Links to the CMS letters of April 2021 at: Kaiser Family Foundation. Work Requirement Waivers: Approved and Pending as of June 28, 2021, available at: https://www.kff.org/medicaid/issue-brief/medicaid-waiver-tracker-approved-and-pending-section-1115-waivers-by-state/#Table2.

\# The program was ended by newly elected Governor in December 2019.

^ Copies of all certification documents and associated materials, insofar publicly accessible, available from the authors.

© 2021 Schmidt H, et al. *JAMA Health Forum.*

eMethods: Sample survey instrument and description

Below is a complete survey package for one of the 4 states (Arkansas). After the cover letter (identical for all recipients) is a full version of the instrument, with the vignette presenting a patient with severe depression who has been seeing the physician for 2 years, followed by the information materials that were provided (as noted in the instrument, these were printed on colored paper.) The wording of the 4 vignettes is otherwise reproduced below:

Short duration/minor depression
James is a 40-year old man who comes to see you for the first time as a new patient to your practice. He reports that he has been feeling depressed over the past 3 months. He has lost interest in his hobbies and says he has little energy. Though he is down, he continues to socialize with his friends to try to feel better.

Short duration/major depression
James is a 40-year old man who comes to see you for the first time as a new patient to your practice. He reports that he has been feeling depressed over the past 3 months. He has lost interest in his hobbies, regularly has trouble sleeping and concentrating, lost considerable weight, says he has very little energy and that his appetite is poor. He had a similar episode a year ago. You ask him about functional impairment, and he says he feels worthless and is unable to work or look for work or perform any of the other elements of community engagement.
He denies other symptoms.

Short duration/minor depression
James is a 40-year old man whom you have seen as a patient in your practice for the past 2 years. He reports that he has been feeling depressed over the past 3 months. He has lost interest in his hobbies and says he has little energy. Though he is down, he continues to socialize with his friends to try to feel better.

Short duration/major depression
James is a 40-year old man whom you have seen as a patient in your practice for the past 2 years. He reports that he has been feeling depressed over the past 3 months. He has lost interest in his hobbies, regularly has trouble sleeping and concentrating, lost considerable weight, says he has very little energy and that his appetite is poor. He had a similar episode a year ago. You ask him about functional impairment, and he says he feels worthless and is unable to work or look for work or perform any of the other elements of community engagement. He denies other symptoms.

© 2021 Schmidt H, et al. *JAMA Health Forum.*

Perelman School of Medicine
UNIVERSITY *of* PENNSYLVANIA

October 21, 2019

Dr. NAME SURNAME

- By fax -

Dear Dr. NAME,

Arkansas Primary Care Providers' views on Medicaid work and community engagement rules

We are researchers from the University of Pennsylvania who recently wrote to you with an invitation to let us know what work requirement/community engagement policy in Medicaid means for you, your patients and your practice.

We have already heard from many of your colleagues, but not from you. We are writing again because of the importance that your response makes for accurate survey results. It is only by hearing from nearly everyone in the sample that the findings can be representative of Arkansas primary care providers' views, and help robustly inform the ongoing legal challenges around work and community engagement rules.

<u>This survey should take about **5 minutes** to complete</u>.

To respond, please either locate the envelop that should have a little over a week ago and mail back the survey in the pre-stamped envelope. If you prefer to respond online, please visit: https://tinyurl.com/WorkARsurvey (or scan the QR code below). Please use access code: **ADD, KEEP IN BOLD FONT**

Your answers to the survey will be confidential and all data will be reported anonymously only. This study is funded by the Robert Wood Johnson Foundation and has received approval from the University of Pennsylvania Institutional Review Board (Protocol # 32562).

Please email or call us with any questions or concerns. If you do not wish to participate, please return the blank questionnaire in the enclosed envelope.

We completely realize that your time is scarce, but very much hope that you will be able to help us with this important research. We thank you for considering this request,

sincerely

Harald Schmidt, PhD
215-573-4519
schmidth@mail.med.penn.edu

David Grande, MD
215-573-3804
dgrande@pennmedicine.upenn.edu

Penn Institutional Review Board: 215.573.2540, irb@pobox.upenn.edu

Note: Dollar bill included here intentionally.CODE HERE

© 2021 Schmidt H, et al. *JAMA Health Forum.*

Arkansas' Medicaid Work and Community Engagement Requirements

Within the Arkansas Works program, the state recently implemented a policy that requires adults who gained Medicaid coverage through the Affordable Care Act to meet new work and community engagement requirements. The Federal Government permitted these requirements to test new ways of promoting "better mental, physical and emotional health… [and to] help individuals and families rise out of poverty and attain independence" (CMS, SMD: 18-002).

Individuals covered by the policy must report at least 80 hours per month of work, job training, education, job search or job search training (up to 39 hours/month), health education classes (up to 20 hours annually), or community volunteering. If they do not, for any 3 out of 12 months, they lose Medicaid coverage for the remainder of the year. Individuals who are exempt from the requirement include those with substantial caring obligations, those who are pregnant, disabled, or medically frail.

Additional policy details are available on the attached Arkansas Works *information sheet: gray colored* Appendix *A. Further information on the role of providers in determining exemptions: yellow colored* Appendix B.

Due to legal challenges the policy is currently inactive. The United States Court of Appeals for the District of Columbia scheduled an appeal hearing for the fall.

1. Based on the information you have received from the state and other sources before receiving this survey, in general, **how informed do you feel about your role in determining whether patients should be exempt** from work and community engagement requirements? I feel…

   □ fully informed
   □ sufficiently informed
   □ somewhat informed
   □ insufficiently informed
   □ fully uninformed

2. Now we would like you to **consider the following clinical scenario** (which presumes that the Federal and state governments reverse the current legal challenges, as they intend to):

   > James is a 40-year old man whom you have seen as a patient in your practice for the past 2 years. He reports that he has been feeling depressed over the past 3 months. He has lost interest in his hobbies, regularly has trouble sleeping and concentrating, lost considerable weight, says he has very little energy and that his appetite is poor. He had a similar episode a year ago. You ask him about functional impairment, and he says he feels worthless and is unable to work or look for work or perform any of the other elements of community engagement. He denies other symptoms.

   James would like help with how he is feeling but also tells you he found out he will lose his Medicaid coverage in 3 weeks because he has not met the new work and community engagement requirements. His friend mentioned that he could get an exemption from the policy and gave him an information sheet (see gray colored Appendix A).

CODE HERE

James asks if you would help him request an exemption from the work requirement policy. This would require (a) becoming a registered reporter (see yellow colored Appendix B), (b) signing a 1-page authorization form together with the patient, and (c) entering the exemption request online, so that Arkansas Works can consider it, and ultimately decide on the exemption.

2a. Would you **agree to the patient's request** and recommend that he receive an exemption based on medical frailty?

□ Yes □ No

2b. Based on the clinical scenario, and setting aside what administrative effort is required of you, **how appropriate would it be for this patient to receive an exemption** from the work and community engagement requirements?

□ completely appropriate
□ somewhat appropriate
□ neutral
□ somewhat inappropriate
□ completely inappropriate

2c. If the patient ultimately **receives no exemption and returns to your practice**, you would:

□ continue to see the patient and absorb the cost
□ continue to see the patient but try to recover the cost from him
□ continue to see the patient but try to recover the cost in some other way
□ not see the patient but actively help him find another (non-Medicaid, safety net) provider
□ not see the patient suggest he finds another (non-Medicaid, safety net) provider
□ something else:

2d. In your opinion, the **administrative effort** for physicians assisting patients seeking exemptions is:

□ completely appropriate
□ somewhat appropriate
□ neutral
□ somewhat inappropriate
□ completely inappropriate

CODE HERE

3. If work and community engagement requirement became permanent, in your own practice, **how likely is it** that you will be faced with patients who come to see you for health care needs, but have either lost coverage because they did not meet requirements, or are about to, as in the above scenario?

   □ very likely
   □ likely
   □ unsure
   □ unlikely
   □ very unlikely

4. Please indicate whether, **overall, you approve or disapprove** of Arkansas' work and community engagement requirement, introduced at the beginning of this survey:

   □ strongly approve
   □ somewhat approve
   □ neutral
   □ somewhat disapprove
   □ strongly disapprove

5. Do you have **any other comments** on the questions above, or other aspects of work or community engagement requirements for Medicaid patients that you would like to highlight, such as **how they impact you, your practice or patients**?

Please turn over for a few demographic questions.

CODE HERE

Demographic Questions

1. What is your gender?
□ Male □ Female

2. Please enter your age: ______years

3. Please select your race:
□ White □ Black, African American □ Asian □ American Indian □ Other
□ I prefer not to say

4. Are you of Hispanic, Latino or Spanish Origin?
□ Yes □ No □ I prefer not to say

5. What is your political affiliation?
□ Democrat □ Republican □ Independent □ Other □ I prefer not to say

6. Please state the year you graduated from medical school ________

7. Please select your primary specialty
□ Internal Medicine □ Family Medicine □ General Practice □ Other

8. What percentage of your patients would you estimate is covered through Medicaid?
______%

9. Thinking about the practice you work at most, about how many physicians work in your practice?
□1 (solo) □2-10 □11-50 □50+

Thank you very much for your participation. If you have any other comments about this study or about the care you provide, please share these here/on reverse:

If you would like to receive the study findings, please enter your email address at: https://tinyurl.com/emailsAR

Completing and returning this survey will serve as your consent to take part in this research study, and authorizes the research team to publish submitted information in anonymous form, only.

<u>Please **insert ONLY the cover letter** in the return envelope (no postage required)</u>

CODE HERE

What You Need To Know About the Work Requirement

You are getting this notice because you have Arkansas Works health insurance. Some people who get Arkansas Works have to participate in work activities to keep their insurance. Those people will have to report work activities to DHS. During 2018, this does NOT apply to anyone who is:

- 29 or younger
- 50 or older

For everyone else: DHS decides if you have to report work activities. DHS needs some information from you to decide that. This notice explains how to report work activities and what kinds of activities DHS accepts.

Section 1: What if I receive both SNAP and Arkansas Works benefits?

If you have a work requirement for SNAP and Arkansas Works, you can report online at www.access.arkansas.gov or report to the SNAP program.

Section 2: How do I know if I have to report work activities to DHS?

Are any of these statements true for you?

- I get TEA Cash Assistance.
- I get Unemployment Benefits.
- I work at least 80 hours per month at Arkansas minimum wage.
- I have a disability (includes blindness) or need help with daily living activities.
- I have a child under 18 in my home.
- I am pregnant or was pregnant within the last 60 days.
- I care for a person who cannot care for him/herself.
- I can't work or look for work because of a short-term disability.
- I am in an alcohol or drug treatment program.
- I go to school, vocational, or job training full time.

If any statement is true, then you may have an "exemption" and may not have to report work activities. However, you MUST tell DHS which statement applies to you. Section 3 tells how to do this.

If none of the statements are true, then you MUST report work activities every month. Section 4 tells how to do this.

NOTE: All adults in your household who get Arkansas Works should read these statements to see if they must report work activities. Remember: during 2018, if you are 29 or younger OR you are 50 or older, this does not apply to you. You do not have to do anything.

Section 3: If I think I have an exemption, what should I do?

If you think you have an exemption, you must tell DHS which statements in Section 2 are true for you.

- Report this online at Access Arkansas at www.access.arkansas.gov. See Section 8 about how to use Access Arkansas.
- After you make the report, DHS sends a letter about what to do next. Always read all mail from DHS as soon as you get it.

Section 4: I do not have an exemption. What do I need to do?

If you are not exempt (see Section 2), you must do 80 hours of work activities **each month** to keep Arkansas Works insurance. Report this online at Access Arkansas at www.access.arkansas.gov. If you need help finding a work activity, see the information at the end of this notice. You can combine hours from any of the work activities below to make 80 hours:

Work Activity	Work Activity Hours
Working at a job that earns you money. Report your income each month. DHS will decide your work hours based on the Arkansas minimum wage.	• Your reported income divided by Arkansas minimum wage = work activity hours
Going to school, job training, vocational, or other educational program.	• English As a Second Language (ESL): 1 hour of instruction = 2.5 work activity hours<br>• GED/Basic Skills/Literacy: 1 reported hour = 2 work activity hours<br>• College/University: 1 credit hour = 2.5 work activity hours<br>• High School: 1 hour of instruction = 2.5 work activity hours<br>• Occupational Training: 1 hour of instruction = 2 work activity hours<br>• Unpaid Job Training: 1 reported hour = 1 work activity hour<br>• Vocational Training: 1 credit hour = 2.5 work activity hours
Volunteering in your community. You can find ways to volunteer at www.volunteerar.org.	• 1 reported hour = 1 work activity hour
Looking for a job on your own or going to free job search training at an Arkansas Workforce Center. You may count up to 39 total hours from these activities each month.	• Job Search: 1 reported job contact = 3 work activity hours<br>• Job Search Training: 1 reported hour = 1 work activity hour
Going to a health education class. You may count up to 20 hours each year from this activity. Learn more about these classes at www.access.arkansas.gov. Click on the Arkansas Works button.	• 1 reported hour = 1 work activity hour

Section 5: What is the deadline to report work activities to DHS?

You must report your activities **every month**. The deadline is the 5th day of the next month. For example, you can report January work activities from January 1 to February 5.

Section 6: What happens if I don't meet the work requirement or miss the report deadline?

If you do not report 80 hours of work activities by the deadline each month, you did not meet the work requirement. That makes you "non-compliant" for the month.

If you could not do 80 hours of work activities because of an emergency or serious life-changing event (for example, a natural disaster or hospitalization), you can request a good cause exemption by contacting your local DHS county office.

If you are non-compliant for 3 months in one calendar year (January through December):

- You lose your Arkansas Works insurance.
- Your insurance will stop at the end of your 3rd month of non-compliance.
- You will not be able to get Arkansas Works insurance for the rest of the year (through December).

Even if your situation changes later in the year and you no longer have to report work activities, you will NOT be allowed to get Arkansas Works. You will have to wait until next year to get Arkansas Works. You may still be eligible for Medicaid under another category.

Section 7: What else do I need to tell DHS?

You must report changes to DHS within 10 days after they happen:

- Any change in your life that might affect your eligibility for Arkansas Works.
- Any change for anyone in your household who has Arkansas Works.

Changes You Must Report Within 10 Days	Report Online at Access Arkansas	Report to Your Local Office OR
Contact information changes, including if you move or change your phone number.	Yes	Yes
All income changes, including increases that put you above the limit for your household size.<br>Household Size (Including You) / Monthly Income Limit:<br>1 — $1,396.56<br>2 — $1,893.36<br>3 — $2,390.16<br>4 — $2,886.96<br>5 — $3,383.76<br>Larger households should contact DHS for more information.	Yes	Yes
Someone moves in or out of your household (includes if someone dies).	Yes	Yes
A change in who you file taxes for, like if a child becomes a dependent on someone else's taxes.	Not available online	Yes
A pregnancy in your household ends for any reason, including birth.	Yes	Yes
Anyone in your household is admitted to or discharged from an institution, like a nursing home.	Not available online	Yes
Anyone in your household has a change in their disability status, like being newly approved for disability or their disability ends.	Yes	Yes

Section 8: How do I use Access Arkansas?

To use Access Arkansas:

Arkansas WORKS — **Report Work Activity / Exemption**

1. Go to www.access.arkansas.gov between 7:00 am and 9:00 pm.
2. Answer the voter registration question with 'Yes' or 'No.'
3. Click 'Arkansas Works Log-In.'
4. Click 'Arkansas Works – Report Work Activity / Exemption.' Look for the button!
5. Log in to your online account. If this is the first time to log in, you must create an account. Be sure to save your account user name and password. You will need them each time you log in.
6. Follow the instructions to report your information.

If you need help with Access Arkansas, call 1-855-372-1084 between 8:00 am and 4:30 pm (Monday – Friday). Operators can help, but they cannot do the report for you.

REMEMBER

- **Be sure to read mail from DHS as soon as you get it.**
- Report any changes affecting your Arkansas Works eligibility or changes in your household.
- Do 80 hours of work activities each month.
- Report work activities at www.access.arkansas.gov. You can use any computer with Internet access or lobby computers at your local DHS office. **Do not miss the 5th day of the month deadline!**
- If you do not meet work requirements for any 3 months in a year, you lose Arkansas Works for the rest of the year (through December). This is true even if you meet the requirement later in the year.
- For help with anything in this notice, you can call 1-855-372-1084 or visit a local DHS office.

Free Job Search Help

From the Arkansas Department of Workforce Services
Arkansas Workforce Centers

Because you get Arkansas Works, you can get free job search help from the Arkansas Department of Workforce Services (DWS). DWS has Arkansas Workforce Centers across the state that can tell you about job openings, how to look for a job, training programs, and more.

What kind of help can I get?

If you are unemployed or currently employed and need a better job, DWS Arkansas Workforce Centers can help by providing:

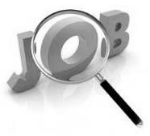

Free Job Search Help:

- Arkansas JobLink lets you post your information and skills for employers to see, search for current job openings, and more. Visit www.arjoblink.arkansas.gov.
- Free computers, telephones, fax machines, and copiers to help you find and apply for jobs.

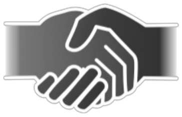

Help from Experts:

- Identify your skills and get help finding job openings that need those skills.
- Create or update your resume to get the best results possible in your job search.
- Career counseling for step-by-step job search guidance.
- Get the facts about what kind of jobs are available and where they are in Arkansas.

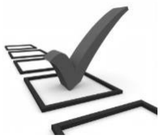

Referrals for Programs for Specific Needs:

- Free GED and free English as a Second Language classes.
- Programs tailored to assist veterans with employment needs.
- Help for workers who have lost a job because of international trade.
- Temporary cash assistance for needy families (TANF/TEA).
- Vocational rehabilitation for Arkansans living with a disability.
- Programs for young people.

How do I get started?

Take the first step today! Contact DWS Arkansas Workforce Centers:

- Online: www.dws.arkansas.gov
- Phone: 1-855-225-4440
- Email: ADWS.Info@arkansas.gov

REGISTERED REPORTER OF WORK AND COMMUNITY ENGAGEMENT ACTIVITIES ACKNOWLEDGMENT FORM

Reporter Information

Reporter Name (*Last, First Middle)*	Relationship to Enrollee/Member	Broker or Agent NPN # *(If Applicable)*<br>Organization or Employer
Reporter Address	Reporter Phone Number	Reporter E-mail Address

☐ **I certify that I have viewed ["Reporting Walkthrough" and "Linking Walkthrough"], a training program that provided information on my role and responsibilities as a Registered Reporter in the Arkansas Works Program.**

- As an authorized Registered Reporter, I am authorized to inspect and/or receive confidential health and work information for the limited purpose of reporting the Arkansas Works (AR Works) work and community engagement activities or exemptions for enrollees/members who have authorized me to enter their data into the portal at www.access.arkansas.gov. I understand that unless authorized by law, I cannot disclose personal information of any enrollee/member to third parties for purposes other than the preparation and reporting of the AR Works work and community engagement activities or exemptions to the DHS Medicaid Program. I understand that enrollees/members may withdraw their consent at any time, and are not required to use my services and I have not made any such representations to enrollees/members.
- I understand that upon receipt of the written revocation I am no longer authorized to access, inspect, and/or receive confidential health and work information concerning the above named enrollee/member or anyone included in the Medicaid file of the enrollee/member.
- I understand that I must electronically report the enrollee's/member's work and community engagement activities and exemptions through the online portal for any enrollees/members who have authorized my services with true, accurate, and correct information, and that I will provide it in accordance with the applicable laws and regulations. I understand that I will not be held responsible for accurately entering information that is provided to me by an enrollee/member that is inaccurate or false. I understand that I may cancel this arrangement with written notice to the enrollee/member and the Arkansas Department of Human Services.
- I acknowledge that the information reported through the portal represents a "claim" that will be presented to the Medicaid program as documentation submitted electronically to justify or help establish or determine what is to be paid for healthcare goods or services delivered to a Medicaid recipient. I understand that knowingly providing false or incomplete information could subject me to legal action, including but not limited to actions brought under the Medicaid False Claims Act or Ark. Code Ann. § 5-36-202 (Theft of Public Benefits).

____
Registered Reporter Signature

____
Date

*This form must be scanned and emailed to AWREPORTER@dhs.arkansas.gov and retained by the registered reporter.

eResults: Non-response bias analysis

We received a total of N=715 complete responses yielding, by American Association for Public Opinion Research standards,[23] a response rate of 21 % (RR1: 20.6%, RR2: 21.2%, RR3: 20.6%, RR4: 21.2%), cooperation rates between 83-85% (CR1: 82.6%, CR2: 84.9%, CR3: 82.6%, CR4: 84.9%), refusal rate of 4% (RefR 1-3 all 3.8%) and a contact rate of 25% (ContR 1-3 all 24.9%). Descriptive statistics on demographic factors for responders are shown in Table 1 of the manuscript.

We provide here three different non-response bias analyses, expanding the data beyond survey response data in that where data were not provided by respondents but were available in the SK&A dataset, missing data were supplemented with SK&A data. For the extent of missing data by variable see Table 1 in the Manuscript. Data that were only provided by responders and that we had no other reference data to compare with are omitted here (race/ethnicity, political affiliation, percent Medicaid population).

In **eTable 2** we compare all responders to all remaining eligible recipients. In **eTable 3**, we compare responders to decliners (i.e., partial responders and those indicating that they wish not to participate). In **eTable 4**, we compare responders to unconfirmed dispositions (those form whom the survey invitation did not bounce back as undeliverable, but who neither responded in full or partially, nor indicated that they would not wish to do so).

By way of a general overview, as per **eTable 2**, we find only modest differences between the sample of responders and all remaining eligible recipients. The overall standardized mean difference in age was 0.1 year, and the overall standardized mean difference in time since graduation was 0.4 years. For categorical variables the difference in proportions between responders and the different types of non-responders never exceeded 4.4% (the largest difference for all demographics for which data on responders and the pool of contacted individuals was available: Gender: 1.2%; Specialty: 4.2%; Physicians in practice: 4.4%; Area Deprivation Index/ADI [of practice location]: 2.4%).

eTable 2: Comparison of responders to all remaining eligible recipients (overall, and by state).

We compare responders answering the vignette prompt, i.e. complete responses (AAPOR category 1: "Complete Interview") to all remaining eligible recipients (i.e., partial responders--excluded from the main analysis, as responder did not complete the vignette, AAPOR category 1 "Partial Interview"); decliners (AAPOR Category 2: "Eligible, non-interview", codes 2.1-2.12); responders unreachable (AAPOR category 3: "Unknown eligibility, non-interview", N=562), and unconfirmed dispositions (AAPOR Category 2: "Eligible, non-interview", codes 2.2-2.27).*

	**Overall (all states)**			**AR**		**IN**		**KY**		**NH**	
**Variable**	Responders (N=715)	Eligible sample (w/o resp.) (N=4031)	SMD or % difference	Responders (N=144)	Eligible sample (w/o resp.) (N=880)	Responders (N=268)	Eligible sample (w/o resp.) (N=1306)	Responders (N=198)	Eligible sample (w/o resp.) (N=1304)	Responders (N=105)	Eligible sample (w/o resp.) (N=541)
**Age (yrs)**											
Mean (SD)	54.1 (12)	54 (12.1)	0.1 (0.1)	55.1 (12.2)	55.1 (12.2)	52.8 (11.8)	52.9 (11.8)	54.5 (12.9)	54.5 (12.9)	55 (10.2)	55 (10.2)
**Graduated (yrs)**											
Mean (SD)	26.7 (12.2)	26.3 (12.3)	0.4 (0.1)	27.6 (12.4)	26.2 (12.9)	25.9 (12.1)	26.3 (12)	27.1 (12.4)	26.4 (12.7)	26.8 (11)	26.4 (10.8)
**Gender**											
Female	242 (33.8)	1396 (34.6)	0.8	39 (27.1)	233 (26.5)	97 (36.2)	479 (36.7)	65 (32.8)	454 (34.8)	41 (39)	230 (42.5)
Male	463 (64.8)	2618 (64.9)	0.1	103 (71.5)	643 (73.1)	165 (61.6)	819 (62.7)	131 (66.2)	845 (64.8)	64 (61)	311 (57.5)
Other/ Declined	10 (1.4)	10 (0.2)	1.2	2 (1.4)	2 (0.2)	6 (2.2)	6 (0.5)	2 (1)	2 (0.2)		
Unknown		7 (0.2)	0.2		2 (0.2)		2 (0.2)		3 (0.2)		
**Specialty**											
Internal	185 (25.9)	1212 (30.1)	4.2	21 (14.6)	180 (20.5)	65 (24.3)	381 (29.2)	66 (33.3)	459 (35.2)	33 (31.4)	193 (35.7)
General	15 (2.1)	72 (1.8)	0.3	7 (4.9)	24 (2.7)	5 (1.9)	15 (1.1)	2 (1)	30 (2.3)	1 (1)	5 (0.9)
Family	515 (72)	2739 (67.9)	4.1	116 (80.5)	674 (76.6)	198 (73.8)	908 (69.5)	130 (65.7)	814 (62.4)	71 (67.6)	343 (63.4)
Unknown		7 (0.2)	0.2		2 (0.2)		2 (0.2)		3 (0.2)		
**PCPs/practice**											
1	147 (20.6)	652 (16.2)	4.4	48 (33.3)	204 (23.2)	44 (16.4)	168 (12.9)	44 (22.2)	243 (18.6)	11 (10.5)	37 (6.8)
2-10	125 (17.5)	695 (17.2)	0.3	22 (15.3)	171 (19.4)	46 (17.2)	258 (19.8)	24 (12.1)	172 (13.2)	33 (31.4)	94 (17.4)
11-50	394 (55.1)	2385 (59.2)	4.1	70 (48.6)	477 (54.2)	152 (56.7)	804 (61.6)	120 (60.6)	780 (59.8)	52 (49.5)	324 (59.9)
50+	49 (6.8)	292 (7.2)	0.4	4 (2.8)	26 (3)	26 (9.7)	74 (5.7)	10 (5.1)	106 (8.1)	9 (8.6)	86 (15.9)
Unknown		7 (0.2)	0.2		2 (0.2)		2 (0.2)		3 (0.2)		
**ADI**											
High	150 (21)	787 (19.5)	1.5	61 (42.4)	280 (31.8)	37 (13.8)	217 (16.6)	48 (24.2)	283 (21.7)	4 (3.8)	7 (1.3)
Medium	317 (44.3)	1687 (41.9)	2.4	65 (45.1)	425 (48.3)	132 (49.3)	547 (41.9)	75 (37.9)	510 (39.1)	45 (42.9)	205 (37.9)
Low	103 (14.4)	742 (18.4)	4	7 (4.9)	82 (9.3)	32 (11.9)	217 (16.6)	13 (6.6)	130 (10)	51 (48.6)	313 (57.9)
Unknown	145 (20.3)	815 (20.2)	0.1	11 (7.6)	93 (10.6)	67 (25)	325 (24.9)	62 (31.3)	381 (29.2)	5 (4.7)	16 (3)

* We exclude ineligible recipients (AAPOR Category 4 "Not eligible", N=124).

eTable 3: Comparison of responders to decliners (overall, and by state).

We compare responders answering the vignette prompt, i.e. complete responses (AAPOR category 1: "Complete Interview") to, partial responders (excluded from the main analysis, i.e responder did not complete the vignette, AAPOR category 1 "Partial Interview") combined with decliners (AAPOR Category 2: "Eligible, non-interview", codes 2.1-2.12).*

	Overall (all states)			AR		IN		KY		NH	
Variable	Responders (N=715)	Decliners and partial responders (N=150)	SMD or % difference	Responders (N=144)	Decliners and partial responders (N=36)	Responders (N=268)	Decliners and partial responders (N=48)	Responders (N=198)	Decliners and partial responders (N=47)	Responders (N=105)	Decliners and partial responders (N=19)
<u>Age (yrs)</u>											
Mean (SD)	54.1 (12)	56.3 (12.1)	2.2 (0.1)	55.1 (12.2)	61.5 (12.2)	52.8 (11.8)	55.3 (10.4)	54.5 (12.9)	52.9 (12.3)	55 (10.2)	57.3 (12.4)
<u>Graduated (yrs)</u>											
Mean (SD)	26.7 (12.2)	28.8 (12.1)	2.1 (0.1)	27.6 (12.4)	34.2 (11.9)	25.9 (12.1)	28.3 (10.6)	27.1 (12.4)	25.3 (12.7)	26.8 (11)	28.5 (11.7)
<u>Gender</u>											
Female	242 (33.8)	52 (34.7)	0.9	39 (27.1)	11 (30.6)	97 (36.2)	14 (29.2)	65 (32.8)	16 (34)	41 (39)	11 (57.9)
Male	463 (64.8)	98 (65.3)	0.5	103 (71.5)	25 (69.4)	165 (61.6)	34 (70.8)	131 (66.2)	31 (66)	64 (61)	8 (42.1)
Other/Declined	10 (1.4)	(0)	1.4	2 (1.4)		6 (2.2)		2 (1)			
<u>Specialty</u>											
Internal	185 (25.9)	50 (33.3)	7.4	21 (14.6)	12 (33.3)	65 (24.3)	13 (27.1)	66 (33.3)	15 (31.9)	33 (31.4)	10 (52.6)
General	15 (2.1)	2 (1.3)	0.8	7 (4.9)	2 (5.6)	5 (1.9)		2 (1)		1 (1)	
Family	515 (72)	98 (65.4)	6.6	116 (80.5)	22 (61.1)	198 (73.8)	35 (72.9)	130 (65.7)	32 (68.1)	71 (67.6)	9 (47.4)
<u>PCPs/practice</u>											
1	147 (20.6)	26 (17.3)	3.3	48 (33.3)	11 (30.6)	44 (16.4)	10 (20.8)	44 (22.2)	4 (8.5)	11 (10.5)	1 (5.3)
2-10	125 (17.5)	14 (9.3)	8.2	22 (15.3)	3 (8.3)	46 (17.2)	3 (6.3)	24 (12.1)	6 (12.8)	33 (31.4)	2 (10.5)
11-50	394 (55.1)	97 (64.7)	9.6	70 (48.6)	21 (58.3)	152 (56.7)	33 (68.7)	120 (60.6)	28 (59.6)	52 (49.5)	15 (79)
50+	49 (6.8)	13 (8.7)	1.9	4 (2.8)	1 (2.8)	26 (9.7)	2 (4.2)	10 (5.1)	9 (19.1)	9 (8.6)	1 (5.2)
<u>ADI</u>											
High	150 (21)	25 (16.7)	4.3	61 (42.4)	15 (41.7)	37 (13.8)	4 (8.3)	48 (24.2)	6 (12.8)	4 (3.8)	0 (0)
Medium	317 (44.3)	62 (41.3)	3	65 (45.1)	16 (44.4)	132 (49.3)	21 (43.8)	75 (37.9)	19 (40.4)	45 (42.9)	6 (31.6)
Low	103 (14.4)	29 (19.3)	4.9	7 (4.9)	4 (11.1)	32 (11.9)	10 (20.8)	13 (6.6)	5 (10.6)	51 (48.6)	10 (52.6)
Unknown	145 (20.3)	34 (22.7)	2.4	11 (7.6)	1 (2.8)	67 (25)	13 (27.1)	62 (31.3)	17 (36.2)	5 (4.7)	3 (15.8)

* We exclude ineligible recipients (AAPOR Category 4 "Not eligible", N=124).

eTable 4: Comparison of responders to unconfirmed dispositions (overall, and by state).

We compare responders answering the vignette prompt, i.e. complete responses (AAPOR category 1: "Complete Interview") to unconfirmed dispositions (AAPOR Category 2: "Eligible, non-interview", codes 2.2-2.27).*

	Overall (all states)			AR		IN		KY		NH	
Variable	Responders (N=715)	Unconf. (N=2605)	SMD or %difference	Responders (N=144)	Unconf. (N=566)	Responders (N=268)	Unconf. (N=812)	Responders (N=198)	Unconf. (N=903)	Responders (N=105)	Unconf. (N=324)
<u>Age (yrs)</u>											
Mean (SD)	54.1 (12)	54 (11.9)	0.1 (0.1)	55.1 (12.2)	54.2 (12.7)	52.8 (11.8)	54.3 (11.4)	54.5 (12.9)	53.7 (12.4)	55 (10.2)	53.9 (10.6)
<u>Graduated (yrs)</u>											
Mean (SD)	26.7 (12.2)	26.3 (12.2)	0.4 (0)	27.6 (12.4)	25.7 (12.8)	25.9 (12.1)	26.8 (11.7)	27.1 (12.4)	26.3 (12.7)	26.8 (11)	26.1 (10.6)
<u>Gender</u>											
Female	242 (33.8)	901 (34.6)	0.8	39 (27.1)	141 (24.9)	97 (36.2)	301 (37.1)	65 (32.8)	317 (35.1)	41 (39)	142 (43.8)
Male	463 (64.8)	1698 (65.2)	0.4	103 (71.5)	423 (74.7)	165 (61.6)	510 (62.8)	131 (66.2)	583 (64.6)	64 (61)	182 (56.2)
Other/Declined	10 (1.4)	6 (0.2)	1.2	2 (1.4)	2 (0.4)	6 (2.2)	1 (0.1)	2 (1)	3 (0.3)		
<u>Specialty</u>											
Internal	185 (25.9)	801 (30.7)	4.8	21 (14.6)	116 (20.5)	65 (24.3)	244 (30)	66 (33.3)	322 (35.7)	33 (31.4)	119 (36.7)
General	15 (2.1)	42 (1.6)	0.5	7 (4.9)	13 (2.3)	5 (1.9)	7 (0.9)	2 (1)	19 (2.1)	1 (1)	3 (0.9)
Family	515 (72)	1756 (67.4)	4.6	116 (80.5)	435 (76.9)	198 (73.8)	560 (69)	130 (65.7)	559 (61.9)	71 (67.6)	202 (62.3)
Unknown	0 (0)	6 (0.2)	0.2		2 (0.4)						
<u>PCPs/practice</u>											
1	147 (20.6)	405 (15.5)	5.1	48 (33.3)	119 (21)	44 (16.4)	100 (12.3)	44 (22.2)	165 (18.3)	11 (10.5)	
2-10	125 (17.5)	415 (15.9)	1.6	22 (15.3)	101 (17.8)	46 (17.2)	158 (19.5)	24 (12.1)	113 (12.5)	33 (31.4)	43 (13.3)
11-50	394 (55.1)	1600 (61.4)	6.3	70 (48.6)	328 (58)	152 (56.7)	523 (64.4)	120 (60.6)	545 (60.4)	52 (49.5)	204 (63)
50+	49 (6.8)	179 (6.9)	0.1	4 (2.8)	16 (2.8)	26 (9.7)	30 (3.7)	10 (5.1)	77 (8.5)	9 (8.6)	56 (17.3)
Unknown	0 (0)	6 (0.2)	0.2		2 (0.4)		1 (0.1)		3 (0.3)		
<u>ADI</u>											
High	150 (21)	496 (19)	2	61 (42.4)	164 (29)	37 (13.8)	137 (16.9)	48 (24.2)	192 (21.3)	4 (3.8)	3 (0.9)
Medium	317 (44.3)	1090 (41.8)	2.5	65 (45.1)	284 (50.2)	132 (49.3)	336 (41.4)	75 (37.9)	356 (39.4)	45 (42.9)	114 (35.2)
Low	103 (14.4)	482 (18.5)	4.1	7 (4.9)	53 (9.4)	32 (11.9)	133 (16.4)	13 (6.6)	95 (10.5)	51 (48.6)	201 (62)
Unknown	145 (20.3)	537 (20.6)	0.3	11 (7.6)	65 (11.5)	67 (25)	206 (25.4)	62 (31.3)	260 (28.8)	5 (4.7)	6 (1.9)

* We exclude ineligible recipients (AAPOR Category 4 "Not eligible", N=124).

eTable 5: Proportional odds model regarding appropriateness of exemption

In this analysis, the outcome is defined ordinally (0 = completely inappropriate; 1 = somewhat inappropriate; 2 = neutral; 3 = somewhat appropriate; 4 = completely appropriate). Further to Table 3 in the manuscript, reporting on the concordance between the attitude of exemption appropriateness and indication of willingness to assist, those finding the administrative effort to be appropriate were estimated to have a 115% higher adjusted odds of reporting a higher degree of exemption appropriateness as compared to those finding the administrative effort to be inappropriate (95% CI: [24.0%, 373%]; p = 0.007.		
**Variable**	**OR (95% CI)**	**p-value**
**Age (years)**	1.02 [0.98, 1.06]	0.28
**Gender**		
Female	--	--
Male	1.11 [0.67, 1.82]	0.69
Other/prefer not to answer	1.58 [0.28, 9.00]	0.61
**Race/Ethnicity**		
Non-Hispanic White	--	--
Non-Hispanic Black	0.34 [0.05, 2.40]	0.28
Hispanic	0.97 [0.47, 2.03]	0.94
Asian	1.84 [0.71, 4.75]	0.21
Other/prefer not to answer	0.62 [0.24, 1.55]	0.30
**State**		
AR	--	--
IN	1.01 [0.55, 1.85]	0.97
KY	0.55 [0.29, 1.03]	0.061
NH	1.72 [0.77, 3.84]	0.19
**Political affiliation**		
Democrat	--	--
Republican	0.20 [0.11, 0.39]	< 0.001
Independent/other	0.35 [0.17, 0.70]	0.003
Prefer not to answer	0.64 [0.34, 1.22]	0.18
**Time since graduated (years)**	0.996 [0.96, 1.04]	0.84
**Specialty**		
Internal	--	--
General	1.01 [0.58, 1.77]	0.97
Family	0.27 [0.07, 1.11]	0.069
Other	1.29 [0.25, 6.72]	0.76
**Percent Medicaid patients (%)**	1.004 [0.99, 1.02]	0.43
**Physicians in practice**		
1	--	--
2+	1.72 [0.93, 3.18]	0.084
11-50	2.28 [1.02, 5.12]	0.046
51+	1.41 [0.48, 4.13]	0.54
**Duration**		
Shorter	--	--
Longer	1.06 [0.66, 1.70]	0.80
**Administrative effort**		
Inappropriate	--	--
Neutral	1.09 [0.64, 1.84]	0.76
Appropriate	2.15 [1.24, 3.73]	0.007
